# Periodontics, Implantology, and Prosthodontics Integrated: The Zenith-Driven Rehabilitation

**DOI:** 10.1155/2017/1070292

**Published:** 2017-06-20

**Authors:** Fausto Frizzera, Mateus Tonetto, Guilherme Cabral, Jamil Awad Shibli, Elcio Marcantonio

**Affiliations:** ^1^FAESA Dental School, Av. Vitória 2220, Vitória, ES, Brazil; ^2^UNIC, Av. Manoel José de Arruda 3100, Cuiabá, MT, Brazil; ^3^Private Practice, Rua Visconde do Rio Branco 649, Taubaté, SP, Brazil; ^4^Department of Periodontology and Oral Implantology, Dental Research Division, University of Guarulhos, Guarulhos, SP, Brazil; ^5^São Paulo State University, Av. Humaitá 1680, Araraquara, SP, Brazil

## Abstract

A customized treatment plan is important to reach results that will satisfy the patient providing esthetics, function, and long-term stability. This type of oral rehabilitation requires professionals from different dental specialties where communication is a major key point. Digital Smile Design allows the practitioners to plan and discuss the patient's condition to establish the proper treatment plan, which must be driven by the desired zenith position. The ideal gingival position will guide the professionals and determine the need to perform surgical procedures or orthodontic movement before placing the final restorations. In this article, the zenith-driven concept is discussed and a challenging case is presented with 4-year follow-up where tooth extraction, immediate implant placement, bone regeneration, and a connective tissue graft were performed.

## 1. Introduction

Multidisciplinary integration is necessary to achieve esthetical and functional results in simple and complex dental rehabilitations. Planning and establishing the correct timing of the involved procedures increase treatment predictability [1]. To perform the oral rehabilitation, it is possible to mimic contralateral teeth form, alignment, and proportion or design it based on esthetical principles and the characteristics of all teeth. Creating a harmonious smile might need interventions from different dental specialties, which will indicate surgical, orthodontic, or restorative procedures [2, 3]. To verify the necessity of such interventions, the gingival contour must be evaluated and establishing the correct gingival zenith aids the treatment planning as well as the following dental procedures.

Gingival zenith is the most apical portion of the gingival margin and is usually distally displaced in maxillary central incisors and centralized in maxillary lateral incisors and canines [4]. Contour of the gingival margins must be in harmony with the smile and facial components so existing alterations or asymmetries require surgical or orthodontic interventions if the patient presents a high lip line or is willing to correct the gingival tissue format. Even after clinical, photographical, and study cast analysis discrete or more complex alterations may not be visualized in the day-to-day practice so a method to boost treatment planning possibilities must be used [5].

Digital Smile Design (DSD) is a planning tool used to facilitate the detection of alterations, individual case planning, and communication between involved personnel [6]. A set of static and dynamic images are acquired from the patient and used to design several reference lines and shapes in the computer to detect alterations and disharmonies. The virtual treatment plan allows that both personnel and patients visualize the main goals and expected results after the treatment as well as its risks and limitations [5]. Furthermore, the communication by digital methods favors treatment sequential procedures as the orthodontist, periodontist, and the restorative team may prospectively progress with the treatment increasing its predictability.

Establishing the ideal position for the gingival zenith with DSD is the first step to recreate a smile. Orthodontic movements are usually indicated whenever it is necessary to perform large horizontal movements in the zenith position [3]. As for smaller horizontal movements and in vertical modifications, periodontal plastic procedures such as crown lengthening or root/implant coverage are good indications [7, 8]. Surgically moving the zenith position coronally with gingival grafts is a more sensitive situation but predictable if properly indicated [9, 10]. This graft also increases the soft tissue volume and prevents gingival or peri-implant tissue recession [11, 12].

Rehabilitation of esthetic areas with implants increased integration between the surgical and restorative procedures. Both treatment phases, performed isolated or concomitantly, have to be in agreement so satisfactory results can be achieved [13, 14]. Whenever a tooth must be removed and replaced by an implant it is important to limit the tissue losses and the collapse of the soft tissue after extraction [15]. If the patient's systemic and local conditions permit, an immediate implant and provisional can be placed in an intact or compromised socket to perform an immediate tooth replacement (ITR) [16–18]. This procedure presents esthetical, psychological, functional, and biological advantages to the patient but must be well indicated in order to achieve treatment success [11, 12, 18, 19]. Despite the limitations and risks reported in the past, ITR combined with bone and gingival grafts present good results maintaining the ridge format and soft tissue contour if the appropriate surgical protocol is employed (Table 1).

Before performing ITR, the ideal zenith position must be established to guide the surgeon in the tridimensional position of the dental implant and in the grafting process. The aim of this paper is to demonstrate an interdisciplinary clinical protocol in order to obtain the better functional and aesthetic results. The protocol was based on the position of the gingival zenith as a starting point and the prediction of the DSD to obtain predictable results.

## 2. Case Description

In this article, a clinical case is reported where, based on a zenith-driven rehabilitation, ITR was performed in a socket with an extensive buccal bone defect, gingival margins from nonadjacent teeth were apically placed, and maxillary anterior teeth received ceramic restorations.

**Figure 1:**
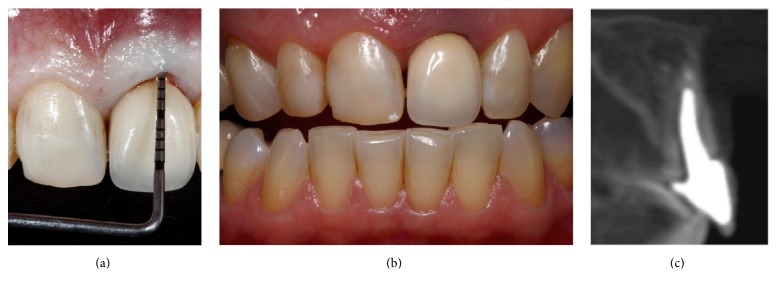
An oblique fracture occurred in tooth 21 due to occlusal trauma. Clinically the patient presented a deep periodontal pocket in tooth 21 (a) thin biotype and shorter clinical crowns in teeth 13 and 12 (b). The buccal bone wall loss and periapical lesion were observed in the CBCT (c).

**Figure 2:**
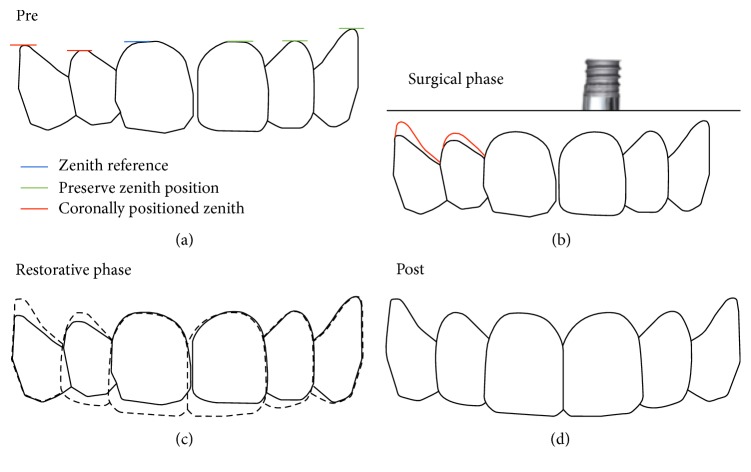
The evaluation of the maxillary anterior teeth demonstrated the alteration of teeth form, zenith position of teeth 13 and 12, and the need to preserve the marginal tissue on tooth 21 after ITR.

A Caucasian 61-year-old female patient with a thin gingival biotype and a high lip line presented an extensive oblique fracture of tooth 21. Clinically there was a marked mobility of the fragment and an increased pocket depth on the buccal aspect (Figures 1(a) and 1(b)). Soft tissue cone beam computed tomography (CBCT) was performed as earlier described [20] and the analysis demonstrated a thin gingival tissue and a marked curve of the metallic post with an oblique fracture that reached the apical third of the tooth. There was proper quantity of palatal bone to place a narrow implant, regardless of the complete loss of the buccal bone wall and the periapical lesion (Figure 1(c)). The patient was unsatisfied with her smile due to tooth form alterations and gingival asymmetry (Figure 2).

**Figure 3:**
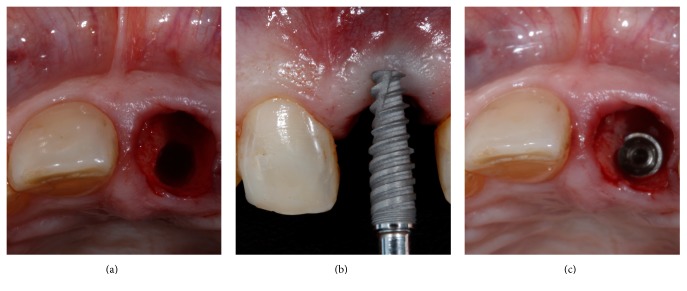
After atraumatic tooth extraction the palatal bone was drilled (a) and a 3.5 × 13 mm was installed (b) 4 mm below the buccal gingival margin (c).

The tooth was gently extracted with a delicate and flexible periotome (Maximus, MG, Brazil) and the socket was cleansed and inspected to confirm the extensive buccal defect. Sequential drilling was performed in the palatal bone (Figure 3(a)) to install a 3.5 × 13 mm implant (Figure 3(b)) with a Morse taper connection (Flash; Conexão Sistema de Próteses, SP, Brazil). In order to create an adequate gingival profile, the implant platform was installed 4 mm below the gingival margin and 0,5 mm more distal than the midtooth position; the obtained insertion torque was 50 Ncm (Figure 3(c)). A polyvinyl siloxane impression (Express XT; 3M ESPE, USA) of the implant position was taken to create a gypsum cast to fabricate a platform switched screw-retained provisional with a concave subgingival contour that was installed 24 hours after the surgery. The sockets were irrigated and inspected to eliminate any particles of the impression material.

**Figure 4:**
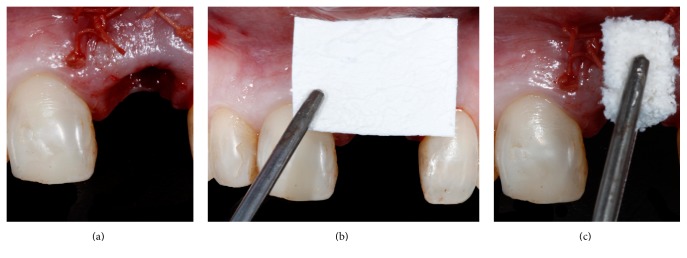
A thick connective tissue graft was sutured internally in the socket buccal tissue (a). A non-cross-linked membrane was trimmed and placed in contact with the soft tissue graft and the most apical portion of the socket (b). The socket was reconstructed with inorganic bovine bone graft containing 10% porcine collagen (c).

**Figure 5:**
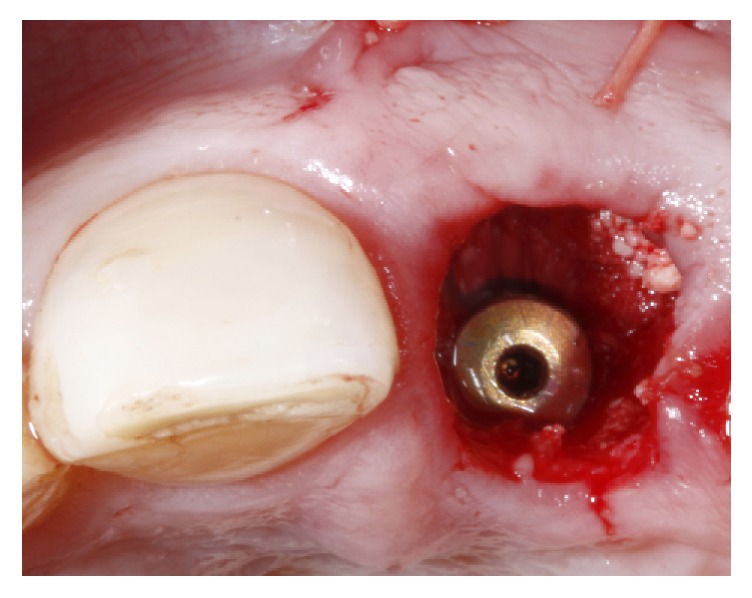
Occlusal view of the reconstructed socket prior to the installation of the provisional with a concave subgingival contour without occlusal contacts.

**Figure 6:**
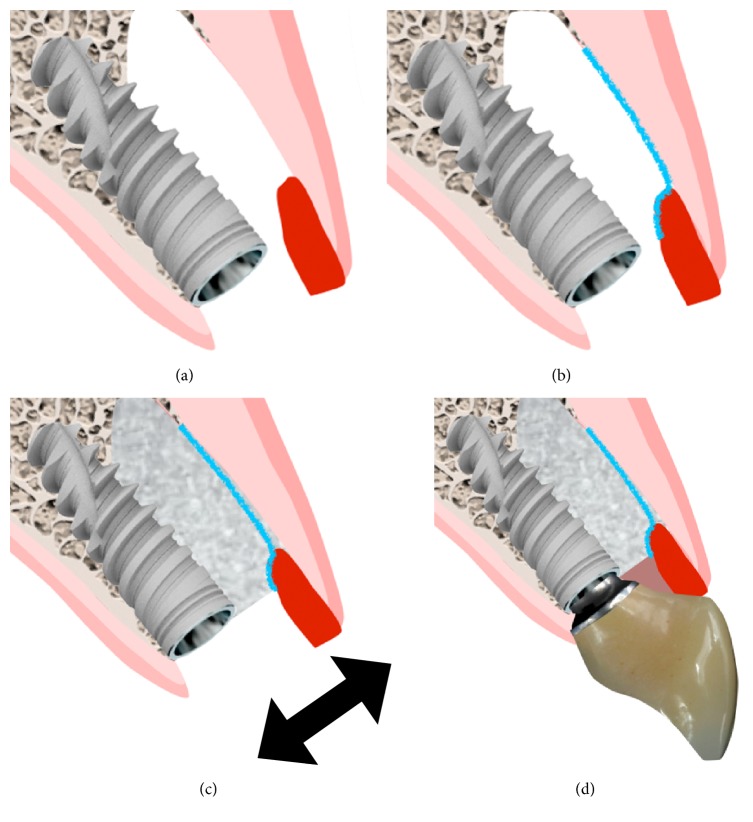
Lateral images demonstrating the sequence (a–d) of the reconstruction of the socket with the grafts and the immediate provisional.

**Figure 7:**
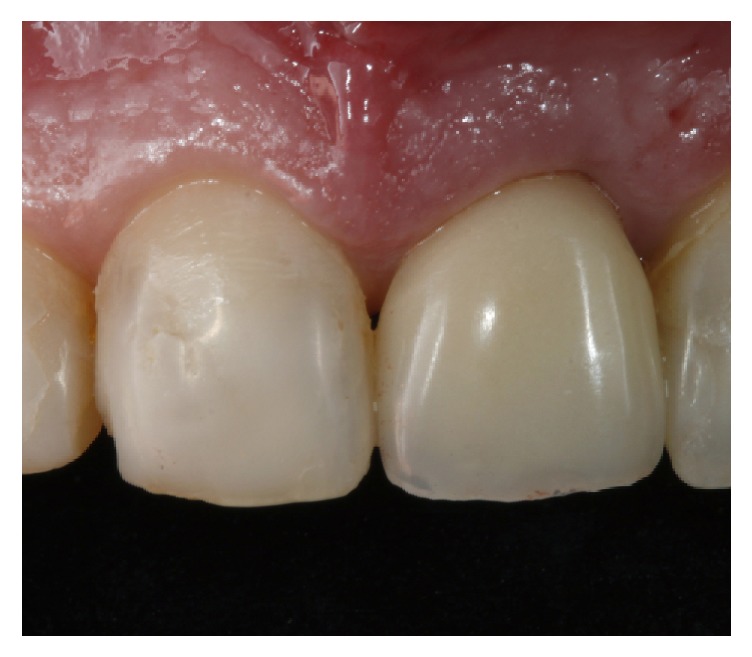
Operated area after 15 days demonstrating the maintenance of the gingival contour.

A buccal pouch was created in the implant facial aspect and a 1.5 mm thick subepithelial connective tissue graft (CTG) was removed from the palate with the single incision technique. The graft was inserted in the pouch and sutured into the buccal aspect of the socket at the level of the gingival margin with a 5-0 Polyglactin 910 suture (Vicryl; Ethicon, Brazil) (Figure 4(a)). Thereafter a non-cross-linked collagen membrane (Bio-Gide; Geistlich Biomaterials, Switzerland) was trimmed according to the bone defect and placed internally to the soft tissue graft (Figure 4(b)). The space between the collagen membrane and the implant was filled with an anorganic bovine bone mineral associated with porcine collagen (Bio-Oss Collagen; Geistlich Biomaterials, Switzerland) (Figure 4(c)). A healing abutment was installed and a provisional restoration was bonded to the adjacent tooth until the screw-retained provisional was fabricated with a tooth shell and titanium UCLA abutment (Figures 5 and 6(a)–6(d)). The provisional was placed in infraocclusion at lateral excursive movements and maximal intercuspal position and in centric occlusion. Postoperatively the patient received 500 mg of amoxicillin per 7 days and 500 mg of paracetamol for 3 days. The patient was also instructed to rinse carefully their mouth with chlorhexidine solution for 14 days and not to brush the area for 5 days. After 15 days the sutures were removed and the gingival tissue presented an adequate form (Figure 7). The patient returned at 30 and 90 days after the surgery.

**Figure 8:**
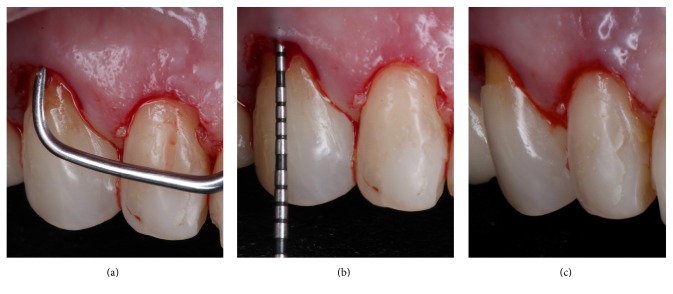
Ninety days after the first intervention a flapless crown lengthening surgery was performed to reposition the zenith in teeth 12 and 13. Bone removal was performed with periodontal Micro-Chisel and curette (a) to establish a 3 mm distance between the gingival margin and bone crest (b and c).

To correct the gingival discrepancy at day 90, flapless crown lengthening of teeth 12 and 13 was performed to level the margin of the opposing teeth 22 and 23 (Figures 8(a)–8(c)). One millimeter of gingival and bone tissue was removed from both teeth without raising a flap. The gingival margins of the other teeth were not manipulated, including the implant placed at the region of tooth 21. At day 180, the implant did not present any clinical alterations and the fabrication of the definitive restorations was initiated. Since there was anatomical alterations and excessive resin material in the anterior teeth, the fabrication of porcelain veneers on teeth 13, 12, 11, 22, and 23 and full porcelain crowns at 14, 21, and 24 was proposed.

**Figure 9:**
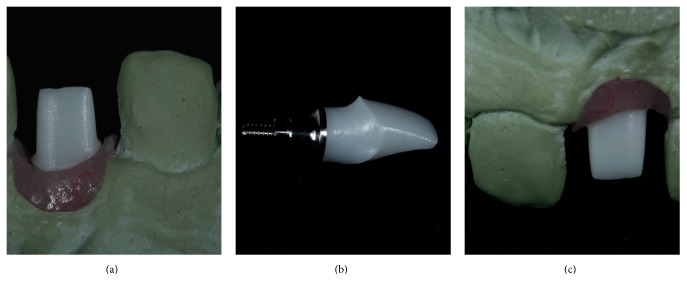
After the osseointegration period, an impression of the implant was taken to fabricate a customized zirconia abutment with a subgingival concave contour (a–c).

**Figure 10:**
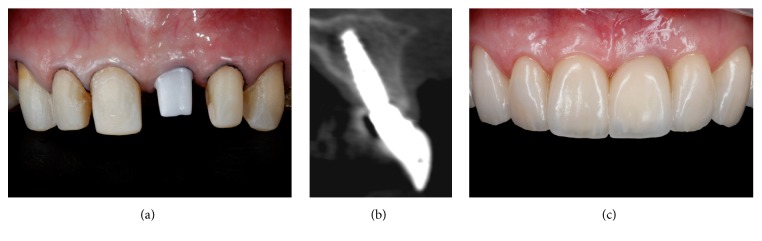
The adjacent teeth were prepared and an impression was made to produce all ceramic restorations (a). A CBCT demonstrated the creation of bone tissue around the implant and the increase of the gingival tissue one year after ITR (b). Results obtained one year after the zenith-driven rehabilitation (c).

The emergency profile was copied from the implant provisional restoration to the transfer coping with a pattern resin and an open-tray impression was performed. A soft tissue/gypsum cast was created and a wax-up of the final custom abutment was designed. The waxed abutment was scanned and a CAD/CAM zirconia custom abutment (Precision; Conexão Sistema de Próteses, SP, Brazil) with a subgingival concave contour was manufactured. After abutment connection (Figures 9(a)–9(c)), teeth 14, 13, 12, 11, 22, 23, and 24 were prepared (Figure 10(a)) and molded with polyvinyl siloxane and a provisional was fabricated with resin (Protemp; 3M ESPE, USA). The porcelain restorations were produced and the veneers were cemented with Rely X Veneer (3M ESPE, USA) and the crowns with Rely X Arc (3M ESPE, USA). For the implant crown, an abutment replica as the previously described technique [21, 22] was utilized to avoid excessive cement in the subgingival region.

**Figure 11:**
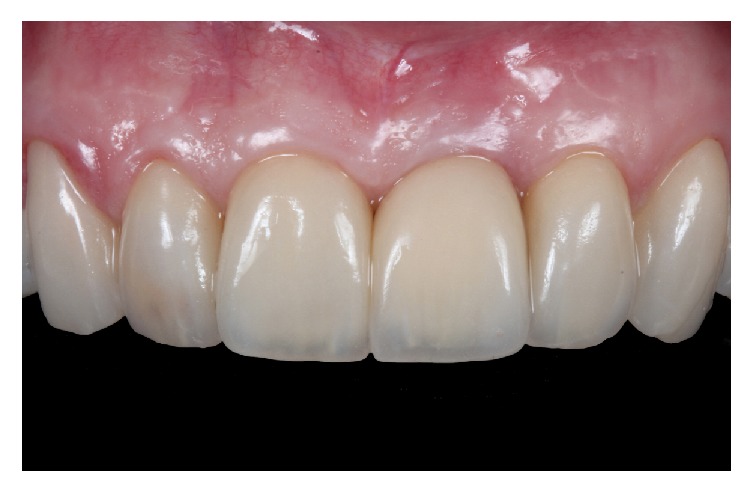
Stable results were obtained after four years of follow-up of the zenith-driven rehabilitation.

A harmonic result was achieved due to the performed treatment protocol (Table 1) and the patient was extremely satisfied. Another soft tissue CBCT (Figure 10(b)) and clinical pictures were performed one year after the surgery where a short-term stability could be seen of the results achieved by the described protocol (Figure 10(c)). The implant presented bone around its surface and there was a complete buccal bone wall with a 3 mm thickness at implant level; the conversion of a thin to a thick biotype could be observed, whereas a 2.5 mm thick gingival tissue was present in the buccal aspect two millimeters below the gingival margin. Four years later, the results were maintained (Figure 11).

## 3. Discussion

It is essential that the professionals involved in treatments that require multidisciplinary procedures work together to achieve the expected results and patient's expectations. Employing DSD in dental rehabilitations aids in the diagnosis process, treatment planning, and communication and visualization of the required procedures by the involved personnel and patients [5, 6].

Establishing the ideal gingival zenith before starting the treatment is important to guide the periodontal, orthodontic, and restorative procedures and also the ideal implant tridimensional position. The rationale of installing the implant anchored in the palatal wall and 4 mm below the buccal gingival margin is to create an adequate emergence profile from the narrow implant platform, increasing the formation/reconstruction of the buccal bone wall to obtain stable results of the hard and soft tissues in the long term [19, 23, 24]. Graft resorption in the horizontal aspect can be equalized by the use of a thick connective tissue graft [25]. The flapless approach with the combination of the bone graft, barrier membrane, and soft tissue graft can be utilized if the zenith position must be stabilized or slightly moved coronally. Whenever indicated immediate tooth replacement must be performed since it reduces treatment time, costs, number of surgeries, and morbidity.

The association of a subepithelial connective tissue graft to the technique promoted the conversion from a thin to a thick biotype and also reduced the apical migration of the tissue margin. Several studies have demonstrated the possibility of minimizing tissue recession when a connective tissue graft is utilized associated with ITR [12, 18, 19]. Minor alterations have occurred in regions that received the soft tissue graft in comparison to nongrafted areas. It is even possible to move the margin coronally when the soft tissue is minimally exposed and there is a concave emergence profile of the abutment/provisional restoration [13, 26]. This procedure also increases the thickness of the buccal soft tissue, which has been demonstrated, which is less prone to facial recession after a long-term follow-up [11, 18, 27].

Since a harmonic zenith position is usually necessary in patients with a high lip line in this clinical case a flapless crown lengthening procedure was also performed in the contralateral maxillary lateral incisor and canine. This kind of procedure has been previously described and demonstrates stable results especially in areas with thin soft tissue [28, 29]. By not reflecting a flap the surgery time and healing of periodontal tissues are also benefited but it is important to carefully remove the excess bone and periodontal fibers creating a new biological width and moving the zenith apically [28, 30].

## 4. Conclusion

Multidisciplinary integration and communication are important to increase treatment predictability. The zenith-driven rehabilitation guides the professionals and allows the visualization of the required procedures to achieve satisfactory results even in complex-high esthetic demanding cases.

## Figures and Tables

**Table 1:** An appropriate protocol is used to perform immediate tooth replacement in sockets with a buccal wall defect.

Preevaluation and planning	Patient medical history; clinical and radiographic analysis of soft and hard tissue quantity/quality to install an immediate implant; DSD planning
Tooth extraction	Gentle tooth extraction
Implant placement	Flapless immediate installation of a narrow implant in a proper tridimensional position
Socket reconstruction	Combination of slow resorbing graft and a non-cross-linked collagen membrane to reconstruct the buccal wall
Soft tissue graft	A thick connective tissue graft increases volume and maintains tissue margin
Immediate restoration	A screw-retained provisional without occlusal contacts and a platform switched concave design is installed
Definitive restoration	Performed after implant osseointegration and soft and hard tissue healing; use of an abutment with the appropriate emergence profile and adaptation
